# Harmonising data collection from osteoarthritis studies to enable stratification: recommendations on core data collection from an Arthritis Research UK clinical studies group

**DOI:** 10.1093/rheumatology/kew201

**Published:** 2016-04-15

**Authors:** Sarah R. Kingsbury, Nadia Corp, Fiona E. Watt, David T. Felson, Terence W. O’Neill, Cathy A. Holt, Richard K. Jones, Philip G. Conaghan, Arthritis Research UK Osteoarthritis and Crystal Disease Clinical Studies Group working group, Nigel K. Arden

**Affiliations:** ^1^Institute of Rheumatic and Musculoskeletal Medicine and NIHR Leeds Musculoskeletal Biomedical Research Unit, University of Leeds, Leeds; ^2^Arthritis Research UK Primary Care Centre, Research Institute for Primary Care and Health Sciences, Keele University, Keele; ^3^Arthritis Research UK Centre for Osteoarthritis Pathogenesis, Kennedy Institute of Rheumatology, Nuffield Department of Orthopaedics, Rheumatology and Musculoskeletal Sciences, University of Oxford, Oxford; ^4^Arthritis Research UK Epidemiology Unit, University of Manchester, Manchester, UK; ^5^Clinical Epidemiology Unit, Boston University School of Medicine, Boston, MA, USA,; ^6^School of Engineering, Cardiff University, Wales; ^7^School of Health Sciences, University of Salford, Manchester; ^8^Arthritis Research UK Centre for Sport, Exercise and Osteoarthritis, University of Oxford; ^9^MRC Lifecourse Epidemiology Unit, University of Southampton, Southampton, UK

**Keywords:** osteoarthritis, clinical trials, stratification, prognosis, personalized medicine

## Abstract

**Objective.** Treatment of OA by stratifying for commonly used and novel therapies will likely improve the range of effective therapy options and their rational deployment in this undertreated, chronic disease. In order to develop appropriate datasets for conducting *post hoc* analyses to inform approaches to stratification for OA, our aim was to develop recommendations on the minimum data that should be recorded at baseline in all future OA interventional and observational studies.

**Methods.** An Arthritis Research UK study group comprised of 32 experts used a Delphi-style approach supported by a literature review of systematic reviews to come to a consensus on core data collection for OA studies.

**Results.** Thirty-five systematic reviews were used as the basis for the consensus group discussion. For studies with a primary structural endpoint, core domains for collection were defined as BMI, age, gender, racial origin, comorbidities, baseline OA pain, pain in other joints and occupation. In addition to the items generalizable to all anatomical sites, joint-specific domains included radiographic measures, surgical history and anatomical factors, including alignment. To demonstrate clinical relevance for symptom studies, the collection of mental health score, self-efficacy and depression scales were advised in addition to the above.

**Conclusions.** Currently it is not possible to stratify patients with OA into therapeutic groups. A list of core and optional data to be collected in all OA interventional and observational studies was developed, providing a basis for future analyses to identify predictors of progression or response to treatment.

Rheumatology key messages

- Stratification of therapy is key to improving OA treatment.
- Development of OA stratification criteria requires harmonized data collection to enable analysis of pooled data.
- We developed a set of core and optional components for data collection across all OA studies.

## Introduction

OA represents a considerable worldwide health and economic challenge [1]. Although OA is a heterogeneous disease driven by a variety of pathophysiologic factors, current therapy selection is largely arbitrary. In general, treatment is aimed at symptomatic relief rather than targeting pathology, and the choice of therapeutic agent is often based on potential toxicity without consideration of likely efficacy. As such, efficacy of these currently available treatments is poor in the majority of people with OA [2]. Stratification of patients towards targeting of commonly used as well as novel therapies will likely improve the range of effective treatment options and their rational deployment in this undertreated chronic disease.

The development of a stratification strategy for OA requires knowledge of both predictors of disease progression to identify patients requiring treatment and predictors of the response to treatment, which together will allow the identification of subsets of patients within which treatments may have improved efficacy. Such data may be gathered prospectively in well-designed interventional and observational studies and retrospectively through *post hoc* analyses of single studies and linking or pooling of study data for meta-analyses. To ensure robust analyses and reliability of results, consistent data collection across studies is essential.

In order to develop appropriate datasets for stratification in OA, our aim was to develop advice on what minimum data should be recorded at baseline in all future OA interventional and observational studies.

## Methods

The Arthritis Research UK Osteoarthritis and Crystal Diseases Clinical Studies Group conducted a literature review of systematic reviews and convened an expert consensus group to consider core data collection to allow *post hoc* stratification analyses to be conducted.

### Literature review

A review of systematic reviews was conducted to identify prognostic factors of OA in general and more specifically for knee, hip and hand OA. Systematic searches were conducted across four electronic databases [Cochrane Library, Embase (OVID), Medline (OVID) and Web of Science] from inception to August 2015. The search strategy (supplementary Table S1, available at *Rheumatology* Online) was designed in OVID Medline using text words and MeSH and combining terms for OA, prognosis and systematic review. For the other databases, search terms were adapted to the search capabilities of the database. Non-English-language articles, letters, comments and editorials were excluded. Evidence was graded according to classifications in the included reviews, which were designated as conflicting, weak/limited, moderate and strong.

### Consensus group discussion

A group of 32 stakeholders, including rheumatologists, physiotherapists, podiatrists, trialists, orthopaedic surgeons, primary care physicians, scientists and patient representatives who have a particular interest in OA, attended a meeting where the findings of the literature review were presented. The panel meeting started with a predefined objective presented by the chair (P.C.). The objective was to develop guidelines to harmonize data collection across all OA clinical studies.

After discussion, it was agreed that development of recommendations for studies should be based on the predetermined principles explained here. Only clinically relevant domains should be included in the core list. These may be different for trials with structural or symptomatic endpoints. Domains would be based on existing recommendations for appropriate domains to be assessed (including those from the OMERACT).

For the data item to be recommended as a core component there should be evidence of either predicting response to treatment or as a risk factor for progression of OA. Where insufficient evidence currently exists, items should not be included in core components, but may be recommended as additional information to be captured at the study team’s discretion. Since an extensive literature review on the tools used to capture each component was not conducted, the use of a set tool would not be recommended. However, potential tools or mechanisms used to capture each component would be suggested. The choice of tool should depend on its extent of validation and psychometric robustness as well as feasibility issues, including costs. The core components should be revised as more data become available, with a maximum of 5 years before the next revision. Items may be generalizable to all anatomical sites of OA or specific to a particular joint. In trials designed with a primary structural endpoint, symptomatic domains should also be measured to assess the clinical relevance of structural change. Recommendations should apply to all types of OA clinical studies, including pharmacological and non-pharmacological interventional trials and observational studies.

The literature summarizing the current evidence for prognostic factors for OA was presented (N.C.). The panel was then prompted to identify the domains that were felt to be important for inclusion in the core data items and these were compiled. Once identified, each domain was discussed and considered in line with the presented evidence to determine whether they fulfilled the criteria for core data, and those with insufficient evidence were excluded at this time. The discussion was separated by joint, with separate consideration of data collection for knee, hip and hand studies. Consensus on inclusion/exclusion of domains was defined where there was 100% verbal agreement from the panel. For each domain included, appropriate tools for assessment were discussed. Domains for which there was a consensus were included in a list of provisional domains. The provisional list was then transcribed and circulated 1 week after the consensus meeting. This was then refined and finalized following an iterative electronic discussion involving the entire panel.

## Results

### Literature review

A total of 35 systematic reviews were identified for inclusion. Thirteen reported on factors associated with structural/radiographic OA progression, 10 on functional/symptom progression and a further 15 on factors affecting outcomes of interventions for or associated with OA. These were categorized according to the location of OA, type of progression reported, level of evidence identified by the systematic review and whether an association was found or not.

### Literature on structural progression

#### Knee OA

Eight systematic reviews reported on structural/radiographic progression of knee OA (Table 1) [3–10]. Two also reported on clinical outcomes and are therefore also included with respect to symptom progression. Strong evidence was found for significant associations of the following factors with structural progression: increasing age [3]; presence of generalized/multijoint OA [3, 4]; combined radiographic features including increasing osteophyte score, joint space width (JSW), joint space narrowing (JSN), Kellgren–Lawrence (KL) grade and chondrocalcinosis [3]; varus alignment [3, 6, 10]; baseline pain [10]; anterior cruciate ligament injury [8]; increasing serum hyaluronic acid [4, 10]; high levels of TNF-α [10] and increasing urinary C-telopeptide of type II collagen (uCTX-II) [7]. Strong evidence of no significant association was found for physical/regular sports activity or moderate exercise [3–5], radiological severity at baseline [4], baseline pain [4], quadriceps strength [4] and knee injury [4]. Conflicting evidence was reported for association of BMI, clinical/disease severity, leg length inequality and symptom duration with radiologic progression [3, 4].

**Table 1:** Summary of the literature on structural and symptomatic progression

	Knee OA		Hip OA
Domain	Structural/radiographic progression	Functional/symptomatic progression	Structural/radiographic progression
Age	Predictive^a^ [3]	Predictive^a^ [3]	Predictive [11]
Gender	No association [4]	—	—
BMI or weight		—	No association [12]
Physical activity/regular sports activity	No association [3, 4]	No association [3]	—
Baseline pain	No association [4]; Predictive [10]	—	Predictive [11]
Clinical/disease severity (Lequesne index score ≥10)			Predictive [11]
Generalized/multiple joint OA	Predictive^a^ [3]; Association [4]	Predictive^a^ [3]	—
Combined radiographic features including osteophyte score, JSW, JSN, KL grade, chondrocalcinosis	Predictive^a^ [3]	Predictive^a^ [3]	—
Radiological grade (KL grade 3)	—	—	Predictive [11]
Baseline radiological severity	No association [4]	—	—
JSW at baseline	—	—	Predictive [11]
Femoral head migration	—	—	Predictive [11]; Association [12]
Femoral osteophytes	—	—	Predictive [11]
Acetabular osteophytes	—	—	No association [11]
Bony sclerosis	—	—	Predictive [11]
Heberdon’s nodes	Predictive [10]		
Atrophic bone response	—-	—	Association [12]
Alignment (varus/valgus)	Predictive^a^ (varus alignment) [3] [10]; association [6]	Predictive^a^ (varus alignment) [3]	—
Quadriceps muscle strength	No association [4]	—	—
Knee injury	No association [4]	—	—
Anterior cruciate ligament injury	Association [8]		
Serum hyaluronic acid	Association [4]; Predictive [10]	—	—
TNF-α	Predictive [10]		
uCTX-II	Association [7]		

^a^Progression defined as change in pain, function or deterioration in radiographic features. Factors reported in systematic reviews as having a strong level of evidence for an association or no association with structural or symptomatic progression of knee and hip OA.

#### Hip OA

Three systematic reviews examined the progression of hip OA; progression was based on radiological parameters and/or the need for total hip replacement with no clear differentiation made (Table 1) [7, 11, 12]. Strong evidence was found for significant associations of the following factors with structural and/or symptomatic progression: increasing age [11], radiological grade KL hip grade 3 [11], clinical/disease severity (Lequesne index score ⩾10) [11], smaller JSW at baseline [11], supero-lateral femoral head migration [11, 12], presence of femoral osteophytes only [11], presence of bony sclerosis [11], atrophic bone response [12] and higher baseline hip pain [11]. Strong evidence for no association was found for the following factors: BMI and weight [12] and acetabular osteophytes [11]. Conflicting or limited evidence was reported for association with gender, JSN and uCTX-II with structural progression [7, 12].

#### Hand OA

One systematic review reported on structural/radiographic progression of hand OA [13]. No strong evidence was available for any factors with a relationship to progression. Limited evidence for association of baseline pain, early menopause, nodal OA and erosive OA with radiographic progression was reported [13].

#### General OA

Two systematic reviews reported on structural/radiographic progression of general OA (either hip OA or knee OA or both hip and knee OA), neither of which identified strong evidence for any factors related to progression [14, 15].

### Literature on symptom progression

#### Knee OA

Five systematic reviews reported on functional/symptomatic progression of knee OA (Table 1) [3, 5, 16–18]. Strong evidence was indicated for the association of the following factors with progressionː increasing age [3]; presence of generalized/multijoint OA [3]; combined radiographic features including increasing osteophyte score, JSW, JSN, KL grade and chondrocalcinosis [3]; and varus alignment [3]. Physical activity was not associated with progression [3]. There was conflicting or limited evidence for associations with BMI [3, 17, 18], gender [3, 17, 18], symptom severity [3, 17, 18], mental health score [3, 17, 18], self-efficacy [3, 16, 17], clinical/disease severity [3], co-morbidity [17, 18] and baseline pain [3] with symptomatic progression.

#### Hip OA

There were no systematic reviews that reported on functional/symptomatic progression of hip OA.

#### Hand OA

Two systematic reviews reported on functional/symptomatic progression of hand OA [13, 19]. Kwok *et al.* [13] found no strong evidence available for the relationship of any factors to progression, while Nicholls *et al.* [19] concluded no information was available on the progression of hand pain and function over time. Limited evidence was reported for an association of age, baseline pain, number of painful joints and function with symptomatic progression, while limited evidence for no association with symptomatic progression was reported for nodal and erosive OA [13].

#### General OA

Three systematic reviews reported on functional/symptomatic progression of general OA (hip OA, knee OA or both) [20], chronic musculoskeletal disease [21] and chronic disorders [22]. Strong evidence was indicated for lower self-efficacy as a predictor of disability in general OA [20]. However self-efficacy was not associated with pain in general OA [20].

### Literature on response to interventions

#### Knee OA

Eleven systematic reviews reported on factors affecting outcomes of interventions for or including knee OA (Table 2) [23–33]. Strong evidence was indicated for association of the following factors with symptomatic outcomesː female gender was associated with pain while waiting for total joint replacement [28], worse preoperative mental health score was associated with lower function and greater pain >1 year after total knee arthroplasty (TKA) [25], increased pain catastrophizing was associated with postoperative pain within 1 year after TKA [25], postoperative self-efficacy was associated with short- and long-term outcomes [32] and co-morbidity was associated with TKA outcomes [30]. The following factors were found not to be associated with symptomatic outcomesː preoperative depression and anxiety was not associated with postoperative functioning within 1 year after TKA [25] and wait for surgery (<180 days) was not associated with pain progression [28]. Limited or conflicting evidence was reported for association of age, BMI, baseline pain and pain duration with response to intra-articular steroid injection [23] and for association of BMI, age and health-related quality of life with TKA outcomes [29, 30, 33].

**Table 2:** Summary of literature on response to interventions

Domain	Intervention outcomes (knee OA)	Intervention outcomes (hip OA)	Intervention outcomes (OA general)^a^
Gender	Association with pain while waiting for TJA [28]	Association with pain while waiting for TJA [28]	Association with pain while waiting for TJA [28]
Physical activity/regular sports activity	—	—	
Wait for TJA surgery <180 days	No association with pain progression [28]	No association with pain progression [28]	—
Mental health score	Association with lower function and pain ≥1 year post TKA [25]	—	—
Preoperative depression	No association with postoperative functioning <1 year post-TKA [25]	—	—
Depression	—	—	
Pain catastrophizing	Association with postoperative pain, <1 year post-TKA [25]	—	
Self-efficacy	—	—	Post-TJA self-efficacy associated with short and long-term outcomes [32]
Co-morbidity	Associated with outcomes [30]	Associated with outcomes [30]	

^a^Defined as hip OA, knee OA or both. Factors reported in systematic reviews as having a strong level of evidence for an association or no association with knee and hip and general OA intervention outcomes.

#### Hip OA

Nine systematic reviews reported on factors affecting outcomes of interventions for or including hip OA (Table 2) [12, 23, 27–30, 32, 34, 35]. Strong evidence was found for an association of female gender with pain while waiting for total joint replacement [28]. Co-morbidity was associated with total hip arthroplasty (THA) outcomes [30]. No association was found between wait for surgery (<180 days) and progression of pain or self-reported functioning [28]. Limited evidence was reported for association of age and health-related quality of life with THA outcomes and with response to intra-articular steroid injection [23, 29, 30].

### Consensus group recommendation on structural progression

The recommended core items for inclusion in structural progression studies are outlined in Table 3. Injury is known to be an important risk factor for the onset of OA, but its role in progression is less clear and it was therefore not included in the core list. While collection of biological samples is highly desirable, especially for novel biomarker development, with only uCTX-II, serum hyaluronic acid and TNF-α showing evidence of association, it was agreed that their inclusion as a core item could not be justified based on current evidence. However, where study design and logistics allow, collection of biological samples is encouraged. The following items were not agreed for inclusion in the core components at this time due to insufficient evidence: clinical measures of inflammation, structural response to loading, joint circumference, joint laxity, patient-reported aetiology and patient expectation. Although there was conflicting evidence for an association of structural progression with BMI, including strong evidence for no association with hip OA, it was agreed that this should be included in the core data. Co-morbidity was not examined within any of the structural progression reviews, however, given the reported association of co-morbidity with THA and TKA outcomes and its association with symptom outcomes, it was agreed that this should be collected within the core items. Advice for hand OA was confounded by there being only a single systematic review that identified no strong associations with structural progression. On discussion it was agreed that hand surgery, hand dominance and menopausal age should be collected to inform future analyses.

**Table 3:** Recommended core data collection for OA studies

Structural studies	Essential	Desirable
General (all site)	BMI, age, gender, racial origin, comorbidity, baseline OA pain, pain in other joints, occupation	Physical activity
Knee specific	X-ray grade of target joint (weight-bearing KL as minimum) + osteophytes and chondrocalcinosis, previous knee surgery including arthroscopy, alignment (varus/valgus), weight-bearing long leg X-ray or gait assessment	
Hip specific	X-ray grade of target joint + osteophytes and chondrocalcinosis, morphology (head, ball and socket), previous hip surgery, history of FAI	Leg length discrepancy
Hand specific	X-ray grade of target joint + osteophytes and chondrocalcinosis, previous hand surgery, hand dominance, age at menopause	
Additional data collection for symptom studies		
General (all site)	Mental health score, self-efficacy, depression or anxiety	

FAI: femoro-acetabular impingement.

### Consensus group recommendation on symptom progression

It was agreed that all of the core items recommended for structural progression should be included in the core list for symptom progression. The following additional core items were proposed to be included in symptom progression studies to ensure demonstration of clinical relevance: mental health score, self-efficacy and depression or anxiety (Table 3). The difference between the severity of symptoms and symptom progression was noted. In longitudinal cohorts, symptoms have been found to remain stable over years in many patients with knee OA, although different patterns of symptoms have been described [36, 37]. Further consideration is therefore required to define symptom progression, for example, how to define a patient with worse pain and unchanged X-ray compared with a patient with X-ray progression and unchanged pain.

### Advice on choice of tools for data collection

While these recommendations did not set out to recommend a set tool to capture each domain, to ensure consistency in data collection and thus improve the opportunity for data to be pooled, it is suggested that, where possible, items are captured using validated tools and with reference to other clinical studies that might provide an opportunity for later data pooling. Within the scope of these recommendations, we have therefore provided guidance on potential tools that may be considered during study design, but this does not represent a definitive list. For capturing multisite joint pain, use of a joint pain manikin is suggested, since joint counts do not reflect the distribution of joints. At a minimum, such a manikin should capture the joint region (e.g. hand, foot, ankle), although at the discretion of the investigator further differentiation may be captured (e.g. ball of the foot, mid-foot, hindfoot). In line with current IMMPACT guidelines, an 11-point numerical rating scale with a 1-week recall period is suggested for capturing baseline pain [38]. Other validated questionnaires may also be considered for assessing pain and function, including but not limited to joint-specific scales such as the Knee Injury and Osteoarthritis Outcomes Score, Hip Injury and Osteoarthritis Outcomes Score, WOMAC, Oxford Knee/Hip Score, Australian/Canadian Osteoarthritis Hand Index and Functional Index for Hand Osteoarthritis [39–43]. It is recommended that alignment should be captured at a minimum using a measure of varus/valgus deformity, but where possible, consideration should be made for inclusion of either a weight-bearing long leg X-ray, which would indicate static alignment in the sagittal plane, or gait assessment, to indicate coronal and sagittal plane hip–knee–ankle angles and tibial rotation and, if possible, a measure of anterior–posterior deceleration/acceleration of the centre of mass [44]. Where imaging outcomes are included in studies, X-rays should be captured using published protocols to ensure consistency and a weight-bearing (where appropriate) KL grade assessed as a minimum [45, 46]. It is suggested that physical activity be captured using an accelerometer or combined gyroscopic-based measures where possible. Alternatively, a simple performance-based test, such as the 30 s chair stand test, the 4 × 10 m fast walk test and/or the timed stair test [47], or a patient-reported outcome (PRO) may be considered. Potential PROs include the Physical Activity Scale in the Elderly, International Physical Activity Questionnaire or Tegner Activity Scale, or a simple question that captures sedentary versus active lifestyle [48, 49].

## Discussion

For researchers undertaking clinical studies, these recommendations provide an important resource to underpin study design. Furthermore, as new studies are developed in line with these recommendations, a valuable resource will be established to inform future *post hoc* analyses of data pooled from multiple studies. Such analyses may include examining predictors of disease progression to identify patients requiring treatment and predictors of the response to treatment, informing the subsetting of patients within which treatments may have improved efficacy.

The distinction between the recommendations described herein and the work by OMERACT to develop core outcome sets for rheumatologic conditions must be noted. The aim of the OMERACT process is to develop core sets that specify, for each condition, the areas/domains (and associated measurement instruments) necessary to provide the best estimate of benefits of an intervention within the context of a clinical trial or observational study [50]. In contrast, the aim of the current recommendations is to harmonize data collection in order to enable pooling of data (using domains derived from the existing literature, which were influenced by the OMERACT OA core set) for future meta-analyses to examine predictors of response to an intervention and to identify patient phenotypes for stratified therapy. As such, we recommend that these components should be collected at baseline as a minimum, to enable definition of patient subgroups in future analyses, with inclusion in additional study visits at the discretion of the investigators and considered with respect to staff and subject time, expense and applicability to the study in question.

The consensus meeting identified a current relative paucity of data to allow stratification of patients with OA into therapeutic groups, highlighting the need for these recommendations in order to provide a foundation to enable stratified OA treatment. Part of the problem lies in the lack of standardisation of the data collected in clinical trials, with resultant limited ability to pool data from different trials to identify predictors of progression or of response to treatment. The harmonization of data collected as recommended herein will allow these issues to be addressed and enable the treatment of OA to move into the era of personalized medicine. The consensus process identified a number of core components for which there is already some evidence of association with the progression of OA, either at a structural or a symptomatic level. Collection of these components, at baseline as a minimum, provides the starting point for efforts to develop stratification algorithms for OA treatment.

While the current evidence has suggested a number of factors that are not associated with structural and/or symptomatic progression, this does not preclude their inclusion within a study. For example, while injury is known to be an important risk factor for the onset of OA, current evidence suggests that there is no relationship between knee injury and structural progression. However, analyses of association with progression are limited by the use of multiple mechanisms of assessment, and standardisation of individual tools or assessments used may reveal further elements associated with symptom or structural progression for inclusion. Furthermore, the assessment of association of injury with OA progression did not consider factors such as measurement of ongoing joint instability or accurate subanalysis of the type of injury, which may mediate the effect of any such association. Further measures that may be considered include patient-reported measures such as quality of life, serum and urine biomarkers and imaging biomarkers, including US and MRI. Although the latter were not examined within any of the reviews examined herein, with current efforts to develop disease-modifying OA drugs, including agents targeted at specific OA pathologies such as synovitis and bone marrow lesions, such data may prove highly valuable in determining patient phenotypes for individual therapies. Selection of additional components should remain at the discretion of the investigator and reflect appropriateness to the study in question as well as resource issues and patient burden. With inclusion of such components in studies, new evidence will enable further refinement of these recommendations and result in the addition of further domains to the core components.

There are a number of limitations to this work. The systematic literature review only included relevant systematic reviews and meta-analyses relating to the symptomatic and/or structural progression of OA and did not review primary papers or studies examining risk factors for the onset of OA. The dissemination plan was limited to presentation at national meetings and journal publication. The recommendations have not been piloted among users; however, there is a mechanism for updating the recommendations within the Clinical Studies Group framework. Finally, since we were unable to recommend a specific tool for each component, an element of variability will remain among studies designed according to these guidelines. Future work may seek to extend these recommendations with a robust review of the potential tools that may be used to assess each data component so as to improve uniformity further. We are aware of an ongoing EULAR project to detail the available psychometric properties of the commonly used OA outcome measures. Despite these limitations, we believe the work is strengthened by the breadth of stakeholders involved in the consensus process, and most importantly, inclusion of patient and public representation.

In summary, we recommend that in the design of new clinical studies, both interventional and observational, the data components to be captured should be carefully considered in light of these recommendations. Furthermore, care should be taken to include validated tools for all components, and where possible, to consider the design of similar studies that may provide the opportunity for future data pooling. Through considered, informed and unified study design we have the potential to provide a powerful substrate for future studies to underpin stratified treatment for OA. Such stratification of patients with OA may involve the use of clinical criteria, biomarkers or functional markers to target a treatment to a patient that is likely to progress with a phenotype that is driven by the pathway targeted by the treatment under investigation. Novel biomarkers may enable better application of a current treatment or the emergence of a new biomarker/treatment combination that would simultaneously develop a method of stratification and a new treatment. As new biomarkers become available, inclusion in the recommended core data will need to be considered.

## Supplementary Material

Supplementary Data
