# Supplementary material for: Harmonising data collection from osteoarthritis studies to enable stratification: recommendations on core data collection from an Arthritis Research UK clinical studies group
Source: Rheumatology (Oxford). 2016 Apr 15;55(8):1394–402. doi: 10.1093/rheumatology/kew201 (PMC4957675; doi:10.1093/rheumatology/kew201)
Supplement: Supplementary Data [file supp_55_8_1394__index.html]

Harmonising data collection from osteoarthritis studies to enable stratification: recommendations on core data collection from an Arthritis Research UK clinical studies group — Harmonising data collection from osteoarthritis studies to enable stratification: recommendations on core data collection from an Arthritis Research UK clinical studies group — Supplementary Data 

# Harmonising data collection from osteoarthritis studies to enable stratification: recommendations on core data collection from an Arthritis Research UK clinical studies group

## Supplementary Data

files

- Supplementary Data - docx file
