# Supplementary material for: Harmonising data collection from osteoarthritis studies to enable stratification: recommendations on core data collection from an Arthritis Research UK clinical studies group
Source: Rheumatology (Oxford). 2016 Apr 15;55(8):1394–402. doi: 10.1093/rheumatology/kew201 (PMC4957675; doi:10.1093/rheumatology/kew201)
Supplement: Supplementary Data [file supp_kew201_rhe-15-1681-File002.docx]

**Supplementary Data**

**Supplementary table S1. Medline search protocol**

| 1 | exp Osteoarthritis/ |
| --- | --- |
| 2 | osteoarthr*.ti,ab. |
| 3 | OA.ti,ab. |
| 4 | arthrosis.mp. |
| 5 | (degenerative adj (arthritis or joint or joints)).ti,ab. |
| 6 | or/1-5 |
| 7 | exp Prognosis/ |
| 8 | exp Disease Progression/ |
| 9 | prognos*.mp. |
| 10 | predict*.mp. |
| 11 | factor*.mp. |
| 12 | risk*.mp. |
| 13 | model*.mp. |
| 14 | evolution.mp. |
| 15 | history.mp. |
| 16 | indicator*.mp. |
| 17 | course.mp. |
| 18 | rule*.mp. |
| 19 | transition*.mp. |
| 20 | determinant*.mp. |
| 21 | pattern*.mp. |
| 22 | subgroup*.mp. |
| 23 | sub-group*.mp. |
| 24 | screen*.mp. |
| 25 | long-term.mp. |
| 26 | progress*.mp. |
| 27 | modif*.mp. |
| 28 | mediat*.mp. |
| 29 | or/7-28 |
| 30 | exp Epidemiologic Studies/ |
| 31 | cohort*.mp. |
| 32 | follow-up.mp. |
| 33 | ("case control" or "case controlled").mp. |
| 34 | retrospective*.mp. |
| 35 | prospective*.mp. |
| 36 | ((patient* or medical) adj3 (record* or review* or histor*)).mp. |
| 37 | longitudinal*.mp. |
| 38 | inception.mp. |
| 39 | observation*.mp. |
| 40 | time series.mp. |
| 41 | outcome*.mp. |
| 42 | or/30-41 |
| 43 | 6 and 29 and 42 |
| 44 | systematic review.tw. |
| 45 | intervention*.ti. |
| 46 | search*.tw. |
| 47 | review.pt. |
| 48 | meta-analysis.mp. |
| 49 | medline.tw. |
| 50 | or/44-49 |
| 51 | 43 and 50 |
